# Aerobic exercise improves sleep in U. S. active duty service members following brief treatment for posttraumatic stress disorder symptoms

**DOI:** 10.3389/fpsyg.2023.1249543

**Published:** 2023-09-15

**Authors:** Stacey Young-McCaughan, Casey L. Straud, Susannah Bumstead, Kristi E. Pruiksma, Daniel J. Taylor, Vanessa M. Jacoby, Jeffrey S. Yarvis, Alan L. Peterson

**Affiliations:** ^1^Department of Psychiatry and Behavioral Sciences, University of Texas Health Science Center at San Antonio, San Antonio, TX, United States; ^2^Department of Psychology, University of Texas at San Antonio, San Antonio, TX, United States; ^3^Institute for Studies of Religion, Baylor University, Waco, TX, United States; ^4^Department of Psychology and Neuroscience, Baylor University, Waco, TX, United States; ^5^Department of Psychology, University of Arizona, Tucson, AZ, United States; ^6^Department of Behavioral Health, Carl R. Darnall Army Medical Center, Fort Hood, Killeen, TX, United States; ^7^Research and Development Service, South Texas Veterans Health Care System, San Antonio, TX, United States

**Keywords:** aerobic exercise, insomnia, sleep, posttraumatic stress disorder, PTSD, active duty military personnel, lifestyle intervention

## Abstract

**Introduction:**

Physical exercise is a lifestyle intervention that can positively impact aspects of physical and psychological health. There is a growing body of evidence suggesting that physical exercise, sleep, and PTSD are interrelated. This study investigated possible relationships. Three research questions were posed: (1) Did randomization to an aerobic exercise intervention reduce insomnia more than being randomized to an intervention without exercise, (2) Did change in sleep predict change in PTSD symptoms, and (3) Did change in sleep impact the relationship between exercise and PTSD symptom reductions?

**Methods:**

Data were collected from 69 treatment-seeking active duty service members with PTSD symptoms randomized into one of four conditions; two conditions included aerobic exercise, and two conditions did not include exercise. Participants in the exercise groups exercised five times per week keeping their heart rate > 60% of their heart rate reserve for 20–25 min.

**Results:**

At baseline, 58% of participants reported moderate or severe insomnia. PTSD symptom severity decreased following treatment for all groups (*p* < 0.001). Participants randomized to exercise reported greater reductions in insomnia compared to those in the no exercise group (*p* = 0.47). However, change in insomnia did not predict change in PTSD symptoms nor did it significantly impact the relationship between exercise and PTSD symptom reductions.

**Discussion:**

Adding exercise to evidence-based treatments for PTSD could reduce sleep disturbance, a characteristic of PTSD not directly addressed with behavioral therapies. A better understanding of exercise as a lifestyle intervention that can reduce PTSD symptoms and insomnia is warranted.

## Introduction

Upwards of 70% of individuals across the world are exposed to psychological trauma at some point in their lives to include sexual violence, physical attack, an accident, threat with a weapon, natural disaster, or combat (Kessler et al., 1995, 2017). While the majority of individuals go on to live without long-lasting effects, 4–8% develop posttraumatic stress disorder (PTSD; Goldstein et al., 2016) which includes persistent intrusions, avoidance of trauma-related stimuli, negative alterations in cognition and mood, and marked alterations in arousal and reactivity (American Psychiatric Association, 2022) that disrupt their and their loved ones’ quality of life (Schnurr et al., 2009). In service members exposed to military combat, 4–17% develop PTSD (Richardson et al., 2010; Judkins et al., 2020; Krull et al., 2022).

Sleep disturbance, specifically disturbing dreams of the stressful experience and trouble falling or staying asleep, is one of the characteristics of PTSD. In the general population, approximately 70% of people with PTSD also report sleep disturbance (Ohayon and Shapiro, 2000). In service members seeking treatment for PTSD, 70% to 90% report insomnia and 69% to 79% nightmares (Pruiksma et al., 2016; Taylor et al., 2020). Service members with PTSD report sleeping less than 5-h per night on workdays and less than 6-h per night on days-off (Taylor et al., 2020). Sleep disturbance is also prevalent in the veteran population with over 90% of post 9/11 veterans with PTSD entering Veterans Administration Healthcare systems screening positive for insomnia disorder (Colvonen et al., 2020).

The most effective and studied treatments for PTSD are Prolonged Exposure (PE; Foa et al., 2018; Peterson et al., 2021, 2023) and Cognitive Processing Therapy (CPT; Resick et al., 2015, 2017; Peterson et al., 2021, 2022). PE and CPT are typically administered as 60–90 min weekly sessions delivered over several months, although massed (i.e., daily) treatment formats have proved to be just as effective in reducing symptoms with decreased attrition rates from treatment (Foa et al., 2018; Galovski et al., 2022; Held et al., 2022; Peterson et al., 2023).

While sleep disturbance contributes to the diagnosis of PTSD, neither PE nor CPT directly addresses sleep concerns. Sleep disturbances reported prior to the start of treatment are associated with poorer PTSD treatment outcomes following treatment (Taylor et al., 2020; Pruiksma et al., 2023). Following PTSD treatment, service members demonstrate some improvements in sleep, yet a majority continue to report clinically elevated insomnia (74–80%) and nightmares (49–55%; Pruiksma et al., 2016).

Both observational and intervention studies have shown reductions in PTSD symptoms with physical exercise. Although exercise is not an evidence-based treatment for PTSD, five meta-analyses of 39 unique studies testing various forms of exercise alone or in combination with behavioral therapy demonstrated reductions in PTSD symptoms with small to medium effect sizes (Rosenbaum et al., 2015b; Whitworth and Ciccolo, 2016; Oppizzi and Umberger, 2018; Hegberg et al., 2019; Björkman and Ekblom, 2022). Across these studies the type, intensity, duration, frequency, and timing of exercise varied widely and yet consistently individuals who exercised reported decreased PTSD symptoms. Since the mechanism by which exercise addresses PTSD symptoms has not been elucidated, there may be many (see Hegberg et al., 2019 for a review). Exercise may be an *in vivo* exposure with a predictable recovery from exercise-induced tachycardia and sweating, something that feels unpredictable with PTSD-induced alterations in arousal and reactivity. Exercise may enhance neuroplasticity and with improved cognitive function individuals benefit as they engage with the traditional cognitive behavioral therapies. Exercise may normalize the release and metabolism of stress hormones of the hypothalamic pituitary axis resulting in reductions in PTSD symptoms. However, the benefits of exercise for PTSD may only be realized while the individual continues to exercise regularly. Unlike cognitive behavioral therapy, exercise does not provide tools to make sense of the trauma experienced or how to operate in the world without avoiding situations that trigger a recurrence of symptoms.

Exercise has also been shown to improve sleep with small to moderate effect across meta-analyses and systematic reviews (Driver and Taylor, 2000; Kredlow et al., 2015; Dolezal et al., 2017; Wang and Boros, 2021; Xie et al., 2021; Ferreira et al., 2023). Findings varied depending upon the type of exercise tested and how sleep was assessed.

Among veterans with PTSD, observational research showed that regular physical exercise was associated with better sleep quality a year later, but not with a reduction in PTSD symptoms (Bosch et al., 2017). In other observational longitudinal research, sleep quality at baseline was found to mediate the relationship between PTSD symptoms and change in exercise one-year later (Talbot et al., 2014). Others have identified an interactive relationship between sleep and exercise that shaped recovery from PTSD, but the statistical analyses were significant only for veterans with poor sleep at the start of treatment (Babson et al., 2015). Exercise interventions prospectively tested in patients with PTSD as a stand-alone treatment or combined with a behavioral therapy have documented reductions in PTSD symptom severity and improved sleep quality (Rosenbaum et al., 2015b; Whitworth et al., 2019a,b; Hall et al., 2020; McGranahan and O’Connor, 2021).

Based on this research, an analysis of data previously collected as part of an experimental study was undertaken to further investigate the relationships among aerobic exercise, sleep, and posttraumatic stress symptoms in active duty service members. The original study was designed to determine if the efficacy of the imaginal exposure component of PE for symptoms of PTSD could be improved by adding aerobic exercise (Young-McCaughan et al., 2022). PTSD symptom severity decreased an average of 11.4 points on the PTSD Checklist – Stressor-Specific (PCL-S) following treatment (*p* < 0.0001); however, there were no significant differences between the experimental conditions. The parent study found the Cohen’s *d* effect size for exercise alone to be 0.73, larger than others have found (Rosenbaum et al., 2015b; Whitworth and Ciccolo, 2016; Oppizzi and Umberger, 2018; Hegberg et al., 2019; Björkman and Ekblom, 2022). Based on data suggesting that exercise improves sleep, the objective of this paper was to examine if physical exercise reduced insomnia in this sample and whether any change in insomnia predicted change in PTSD symptoms. Three research questions were posed: (1) Did randomization to an aerobic exercise intervention reduce insomnia more than being randomized to an intervention without aerobic exercise, (2) Did change in insomnia predict change in PTSD symptoms, and (3) Did change in insomnia impact the relationship between exercise and PTSD symptom reductions?

## Methods

### Methods

The methods for the clinical trial that collected these data have been reported elsewhere (Young-McCaughan et al., 2022). In short, the original sample consisted of 72 active duty military members seeking treatment for PTSD symptoms. Inclusion criteria were deployment in support of combat operations following 9/11, scoring greater than 25 on the PTSD Checklist – Stressor-Specific (PCL-S; Weathers et al., 1993), and reporting re-experiencing and avoidance symptoms at least once per week. Participants could be taking psychotropic medications but not benzodiazepines. Participants were judged to be safe to engage in aerobic activity. Those who had undergone exposure therapy within the last year or conveyed suicidal ideation that warranted immediate intervention were excluded from the study.

Participants who met the inclusion and exclusion criteria were randomized into one of four conditions: (1) exercise only, (2) imaginal exposure only, (3) imaginal exposure plus exercise, or (4) no exercise/no exposure therapy (control). Participants met with a member of the study staff five times over 8 weeks and completed homework specific to their intervention group. Participants in the exercise groups were prescribed exercise five times per week keeping their heart rate > 60% of their individually determined heart rate reserve for 20–25 min.

The original clinical trial was approved by the Institutional Review Boards at Brooke Army Medical Center (reviewing for Carl R. Darnall Army Medical Center at Fort Cavazos, formerly named Fort Hood), the University of Texas Health Science Center at San Antonio, and the Uniformed Services University.

For this analysis, three participants who were randomized and treated prior to the addition of the Insomnia Severity Index (ISI) were removed from the analyses. Remaining participants (*n* = 69) randomized into one of the two exercise groups (*n* = 35) or one of the two no exercise groups (*n* = 34) were compared.

### Measures

#### Insomnia Severity Index (ISI)

The ISI (Morin, 1993) is a 7-item self-report measure that assesses perceived severity of insomnia. Each item uses a 5-point Likert type scale from 0 to 4, with higher numbers corresponding to greater insomnia severity. The items sum to produce a total score (range 0–28; 0–7 = *no clinically significant insomnia*, 8–14 = *subthreshold insomnia*, 15–21 = *moderately severe clinical insomnia*, 22–28 = *severe clinical insomnia*; Shahid et al., 2011).

#### PTSD Checklist - Stressor-Specific (PCL-S)

The PCL-S (Weathers et al., 1993) is a 17-item measure of symptoms of the Diagnostic and Statistical Manual *(DSM)-IV*-defined PTSD indexed to military experiences (American Psychiatric Association, 2000). Participants rated how much they were bothered by each symptom over the past week on a scale from “1 = *Not at All*” to “5 = *Extremely*.” Higher scores indicate greater PTSD symptoms. Questions 2 and 13 related to sleep disturbance (nightmares and insomnia) were omitted from the PCL-S total score for analyses because the items are sleep related.

#### Data analytic approach

Analyses included all participants who completed a baseline assessment. We used two linear mixed-effects regression models with repeated measures at pre-and post-treatment to address research questions. To address Research Question 1 regarding sleep improvements on the ISI, we employed a linear mixed-effects regression model that included fixed effects of time (pre-and post-treatment), group (exercise vs. not), and the interaction. The dependent variable of interest for Research Question 1 was sleep disturbances on the ISI. Research Questions 2 and 3 were addressed using a linear mixed-effects regression model with fixed effects of time (pre-and post-treatment), group (exercise vs. not), the covariate pre-post change in sleep (ISI), and respective two-and three-way interactions. The dependent variable of interest for Research Questions 2 and 3 was PTSD symptom severity as measured by the PCL-5. Change in sleep as a predictor of change in PTSD (Research Question 2) was based on the two-way interaction between visit and the ISI pre-post change score covariate. The impact of change in sleep on the relationship between exercise and PTSD (Research Question 3), was addressed by the three-way interaction between visit, group, and sleep. Analyses were completed using SPSS version 28.

#### Participants

Data collected from 69 active duty service members seeking treatment for PTSD were included in this secondary analysis. The three participants who were randomized and treated prior to the addition of the ISI were removed from the analyses. Participants were primarily men (*n* = 63, 91%) ranging in age from 22 to 52 (*M* = 35, SD = 7.2). More than half of participants self-identified as a racial minority (Black, Asian, or other; *n* = 38, 55%) and 14 (20%) reported being of Hispanic or Latino ethnicity. Most were married or living with a partner (*n* = 55, 80%), and most reported education beyond high school (*n* = 49, 71%). Almost all participants (*n* = 68, 99%) served in the Army. The length of military service ranged from 3 to 32 years (*M* = 13 years, SD = 6.8). The majority of the participants were junior noncommissioned officers (NCOs; *n* = 50, 74%). Almost half of the participants (*n* = 30, 44%) served in combat arms positions (e.g., Infantry, Armor, Field Artillery, or Special Forces) while deployed, and half of the participants had deployed three or more times (*n* = 35; 51%). See Table 1.

**Table 1:** Demographic, military service, and symptom characteristics of the sample at baseline.

Characteristic	All (*n* = 69)	Exercise (*n* = 35)	No Exercise (*n* = 34)
Age in years			
Range	22–52	22–48	22–52
Mean ± SD	35 ± 7.2	35 ± 7.5	36 ± 6.9
Sex			
Men	63 (91%)	32 (91%)	31 (91%)
Women	6 (9%)	3 (9%)	3 (9%)
Ethnicity			
Hispanic or Latino	14 (20%)	8 (23%)	6 (18%)
Not Hispanic or Latino	55 (80%)	27 (77%)	28 (82%)
Race			
White	31 (45%)	15 (43%)	16 (47%)
Black	22 (32%)	10 (29%)	12 (35%)
Asian	2 (3%)	1 (3%)	1 (3%)
Other	14 (20%)	9 (25%)	5 (15%)
Marital status			
Never married, separated, divorced, not living with partner	14 (20%)	7 (20%)	7 (24%)
Married or in a relationship and living with a partner	55 (80%)	28 (80%)	27 (79%)
Education			
High school	20 (29%)	13 (37%)	7 (20%)
Some college	40 (58%)	19 (54%)	21 (62%)
4-year college degree	5 (7%)	1 (3%)	4 (12%)
Master’s degree	4 (6%)	2 (6%)	2 (6%)
Military service			
Army	68 (99%)	34 (97%)	34 (100%)
Marine	1 (1%)	1 (3%)	0 (0%)
Years in service			
Range	3–32	3–24	4–32
Mean ± SD	13 ± 6.8	12 ± 6.1	13 ± 7.5
Grade			
Junior NCO (E-4 to E-6)	50 (74%)	26 (74%)	24 (73%)
Senior NCO (E-7 to E-9)	14 (20%)	7 (20%)	7 (21%)
Officers (WO-3 & O-3 to O-4)	4 (6%)	2 (6%)	2 (6%)
Duty while deployed*			
Combat Arms	30 (44%)	15 (43%)	15 (44%)
Combat Support	12 (17%)	8 (23%)	4 (12%)
Combat Service Support	27 (39%)	12 (34%)	15 (44%)
Number of deployments			
1	13 (19%)	7 (20%)	6 (18%)
2	21 (30%)	10 (28%)	11 (32%)
3	18 (26%)	9 (26%)	9 (27%)
4 or more	17 (25%)	9 (26%)	8 (23%)
Time (in years) since last redeployment			
Range	0.1–12.8	0.3–5.3	1.0–9.0
Mean ± SD	3 ± 2.5	2 ± 1.7	3 ± 2.6
Time (in years) since index trauma			
Range	1–26	1–12	1–12
Mean ± SD	6.7 ± 4.99	5.7 ± 3.45	6.4 ± 3.81
PTSD Symptoms (PCL-S score including questions 2 and 13 related to sleep disturbance, i. e., nightmares and insomnia)			
Range	25–72	26–70	25–72
Mean ± SD	46.5 ± 13.03	46.7 ± 12.03	46.2 ± 14.17
Percent who met DSM-IV diagnostic criteria for PTSD*	39 (57%)	19 (54%)	20 (59%)
Insomnia (ISI score)			
Range	4–28	4–27	4–28
Mean ± SD	15.1 ± 5.33	15.0 ± 5.42	15.3 ± 5.31
Percent scoring ≥15 (moderate or severe insomnia)	40 (58%)	19 (54%)	21 (62%)

E, enlisted; ISI, Insomnia Severity Index; M, mean; NCO, noncommissioned officer; O, officer; PCL-S, PTSD Checklist-Stressor-Specific; PTSD, posttraumatic stress disorder; WO, warrant officer; SD, standard deviation. *Examples of Combat Arms duty = Infantry, Armor, Field Artillery, Air Defense Artillery, Special Forces & Combat Flight Crews; Examples of Combat Support duty = Chemical Corps, Engineer, Military Intelligence, Military Police including Security Forces, Signal Corps; Examples of Combat Service Support duty = Quartermaster, Ordinance, Transportation including Convoy personnel, Finance, Chaplain, JAG, Medical including Air Evacuation Crews. **Total severity score ≥ 50 and report of at least 1 intrusion symptom, 3 avoidance symptoms, and 2 hyperarousal symptoms, each present at the level of moderate or higher during the past month.

## Results

At baseline, PCL-S scores ranged from 25 to 72 (*M* = 46.5, SD = 13.03). More than half of the study participants (*n* = 39, 57%) met the *DSM-IV* diagnostic criteria for PTSD (total severity score ≥ 50 and report of at least 1 intrusion symptom, 3 avoidance symptoms, and 2 hyperarousal symptoms, each present at the level of moderate or higher during the past month). At baseline, ISI scores ranged from 4 to 28 (*M* = 15.1, SD = 5.33). More than half of the study participants (*n* = 40, 58%) scored ≥15 indicating moderate or severe insomnia (Shahid et al., 2011).

**Figure 1:**
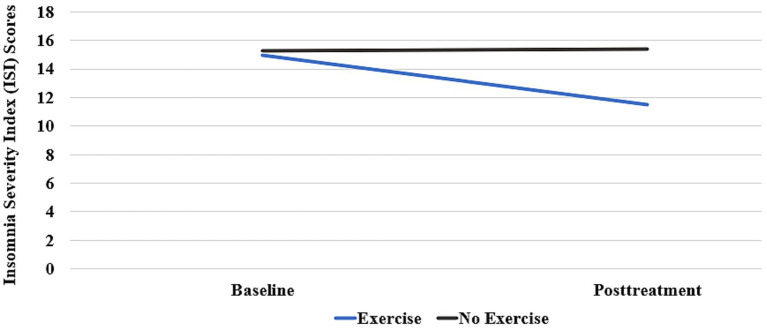
Change over time in estimated marginal means of the Insomnia Severity Index (ISI) between conditions (exercise and no exercise).

Analyses indicated there was a significant interaction between visit and exercise group (*F* (1, 32) = 4.28, *p* = 0.047) showing that those randomized to exercise had greater reductions in insomnia (*M* = −3.41, *p* = 0.014) compared to those in the no exercise group (*M* = 0.17, *p* = 0.884). However, change in insomnia did not predict change in PTSD symptoms (*F* (1, 29) = 1.22, *p* = 0.287) nor did it impact the relationship between exercise group assignment and PTSD symptom reductions [*F* (1, 29) = 1.12, *p* = 0.299]. For the entire sample, there was a significant reduction in PTSD severity over time [*F* (1, 29) = 17.39, *p* < 0.001] regardless of group randomization (exercise and not) after controlling change in insomnia baseline to posttreatment. Those that exercised reported a greater reduction in PTSD symptoms (11.6 point reduction on the PCL-S) compared to those in the no exercise group (8.2 point reduction on the PCL-S), but this was not either a significantly statistical or clinical different between the two exercise groups. See Tables 2, 3 and Figure 1.

**Table 2:** Change over time in estimated marginal means of insomnia (as assessed with the Insomnia Severity Index; ISI) between physical exercise conditions (exercise and no exercise).

Insomnia Severity Index (ISI score)	Baseline	Post	Change
Exercise	15.0 ± 0.91	11.5 ± 1.50	−3.45 ± 1.33
No Exercise	15.3 ± 0.92	15.4 ± 1.34	0.17 ± 1.14

ISI, Insomnia Severity Index. All scores are estimated marginal means using all available data ± standard error. Those randomized to exercise had greater improvements in their sleep compared to those who did not exercise (*M*_diff_ = 3.62, *p* = 0.047).

**Table 3:** Change over time in estimated marginal means of posttraumatic stress symptoms (as assessed with the PTSD Checklist – Stressor-specific; PCL-S) between physical exercise conditions (exercise and no exercise) controlling for change in insomnia severity (as assessed with the Insomnia Severity Index; ISI) baseline to posttreatment.

Posttraumatic stress symptoms (PCL-S score)	Baseline	Post	Change
Exercise	41.2 ± 3.59	29.6 ± 4.49	−11.60 ± 3.19
No Exercise	44.0 ± 2.92	35.8 ± 3.65	−8.20 ± 2.59

PCL-S, PTSD Checklist – Stressor-Specific Version. All scores are estimated marginal means using all available data ± standard error. The relationship between sleep change and change in PTSD symptoms moderated by group randomization (*p* = 0.299). There was a significant reduction in PTSD severity over time (*M* = −9.09, *p* < 0.001). Both groups had significant reductions in PTSD severity after controlling for sleep.

## Discussion

This was a secondary analysis of data collected from a previous study (Young-McCaughan et al., 2022) designed to investigate the possible relationships between aerobic exercise, sleep, and PTSD. To the best of our knowledge, this was the first study to assess change in sleep and PTSD in a randomized clinical study both with and without an exercise intervention in active duty service members.

While other research has found that exercise alone or in combination with behavioral therapy reduces PTSD symptoms compared to a control condition (Rosenbaum et al., 2015b; Whitworth and Ciccolo, 2016; Oppizzi and Umberger, 2018; Hegberg et al., 2019; Björkman and Ekblom, 2022), there was not a differential reduction in PTSD severity over time in this analysis dependent upon randomization to exercise or not. In this study there was a mean reduction of 9 points on the PCL-S (*p* < 0.001). However, unlike previous research that used a no treatment control, participants in the parent study who did not exercise could have received exposure therapy.

Like previous research (Talbot et al., 2014; Babson et al., 2015; Bosch et al., 2017), this analysis found that those randomized to exercise had greater reductions in insomnia compared to those in the no exercise group. The effect size for exercise on the ISI was *d = 0*.73. Of clinical significance in this study is that the mean ISI score at baseline for all participants was 15.1 (SD = 0.91) which is in the moderate clinical insomnia range of 15–21. Following treatment, the ISI in the exercise group decreased to 11.5 (in the subthreshold insomnia range of 8–14) while the no exercise group stayed at 15.4 still in the moderate clinical insomnia range of 15–21. As sleep is disturbed in up to half of service members (Troxel et al., 2015) and up to 92% of service members seeking treatment for PTSD (Pruiksma et al., 2016), the finding that exercise reduced insomnia supports the recommendation for exercise as a behavioral lifestyle intervention for insomnia. Exercise has the added benefits of few side effects and the ability of the individual to exercise without a prescription or need to meet repeatedly with a behavioral health provider.

However, surprisingly, in this analysis the observed change in insomnia in the group that exercised did not predict change in PTSD symptoms. While other research has shown that treating insomnia with cognitive behavioral therapy results in better PTSD outcomes (Taylor et al., 2023), the same was not seen in this study suggesting that it was a mechanism other than reduced insomnia by which exercise reduced PTSD symptoms.

Before exercise can be recommended as an evidence-based intervention for PTSD, we believe that a better understanding of the mechanism by which symptoms are relieved is warranted. Exercise does not afford any guided processing of the traumatic event and so while management of acute PTSD symptoms could be managed by engaging in a strenuous workout, this does not address the underlying disease pathology. Controlled trials with longer 6- and 12-month follow-up assessments are warranted and yet difficult to conduct in that a researcher cannot ethically ask a research participants randomized to a no exercise condition to not exercise for a year or more considering the adverse health effects of sedentary behaviors.

And yet, there are reasons to continue to recommend exercise as a lifestyle intervention adjunct for traumatic stress. The U. S. Department of Health and Human Services (2018) Physical Activity Guidelines for Americans cite numerous health benefits of physical activity specific to psychological health including (1) reduced anxiety and depression, and (2) improved sleep and cognition. Low levels of physical activity are observed in individuals with mental disorders including PTSD (Zen et al., 2012; Rosenbaum et al., 2015a; Firth et al., 2020). Although there is a growing body of evidence suggesting that physical exercise, sleep, and PTSD are interrelated, further research is needed to better understand these relationships to inform treatment.

### Limitations

There are several limitations to this secondary analysis. The primary limitation is not being able to know either how closely the exercise group adhered to their exercise prescription or how much the no exercise group may have exercised outside of the study. The parent study recruited primarily men (*n* = 63, 91% of the sample in this analysis) which reduces the generalizability of the findings. And yet, having data from a large sample of men to evaluate provides unique insight into the experience of men with PTSD, a minority of the patients who develop PTSD compared to women. Another limitation of the parent study was the inclusion of both individuals with only PTSD symptoms in addition to individuals who met full diagnostic criteria for PTSD. Patients not meeting diagnostic criteria for PTSD could differ in their response to treatment and confound the study findings. Likewise, 42% of the study participants (*n* = 29) did not report clinically significant insomnia on the ISI. Although those reporting insomnia were equally randomized into each group, with only 69 participants in the entire sample, the power to detect a mediating effect of sleep on PTSD outcomes may have been too small. The assessment of sleep using only the ISI is another limitation of the study. Of note, at the time the parent study was conducted, the *DSM-5* (American Psychiatric Association, 2022) had not been published. The diagnostic criteria for the *DSM-5* and use of assessments specific to the *DSM-5* should be used in future research.

## Conclusion

This study found that treatment-seeking active duty service members with PTSD randomized to an aerobic exercise intervention reported greater reductions in insomnia compared to those randomized to a non-exercise intervention. However, reductions in insomnia did not predict a change in PTSD symptoms. A better understanding of exercise as a lifestyle intervention that can positively impact aspects of physical and psychological health, including sleep and PTSD, is warranted.

## Data availability statement

The data from this study are maintained at The University of Texas Health Science Center at San Antonio in the STRONG STAR Repository. Requests for access to the data as well as for materials and the analysis code also can be emailed to repository@strongstar.org.

## Ethics statement

The study that collected the data analyzed for this manuscript was approved by the Institutional Review Boards at Brooke Army Medical Center (reviewing for Carl R. Darnall Army Medical Center), the University of Texas Health Science Center at San Antonio, and the Uniformed Services University. The study was conducted in accordance with the local legislation and institutional requirements. The participants provided their written informed consent to participate in this study.

## Author contributions

SY-M, CS, SB, and AP contributed to the conception and design of the study. SY-M, KP, DT, VJ, JY, and AP contributed to its implementation. CS performed the statistical analysis. SY-M and SB wrote the first draft of the manuscript. All authors reviewed the manuscript, read, and approved the submitted version.

## Funding

U.S. Department of Defense through the Uniformed Services University of the Health Sciences TriService Nursing Research Program award HU0001-09-1-TS15 and the Dielmann Distinguished Professorship for Advanced Methods in Psychiatric Research.

## Conflict of interest

The authors declare that the research was conducted in the absence of any commercial or financial relationships that could be construed as a potential conflict of interest.

## Publisher’s note

All claims expressed in this article are solely those of the authors and do not necessarily represent those of their affiliated organizations, or those of the publisher, the editors and the reviewers. Any product that may be evaluated in this article, or claim that may be made by its manufacturer, is not guaranteed or endorsed by the publisher.

## Author disclaimer

The views expressed herein are solely those of the authors and do not reflect an endorsement by or the official policy or position of Brooke Army Medical Center, C.R. Darnall Army Medical Center, the U.S. Army Medical Department, the Defense Health Agency, the U.S. Army Office of the Surgeon General, the Department of the Army, the Department of the Air Force, the Department of Defense, nor any agencies under the U.S. Government.
